# Nanoparticle Contrast Agents for Dark-Field X-ray Imaging

**DOI:** 10.1021/acs.nanolett.4c04878

**Published:** 2024-11-27

**Authors:** Carlos Navarrete-León, Adam Doherty, Margarita Strimaite, Joseph C. Bear, Alessandro Olivo, Marco Endrizzi, P. Stephen Patrick

**Affiliations:** † Department of Medical Physics and Biomedical Engineering, University College London, London, WC1E 6BT, United Kingdom; ‡ X-ray microscopy and tomography lab, The Francis Crick Institute, London, NW1 1AT, United Kingdom; § Centre for Advanced Biomedical Imaging, Division of Medicine, University College London, London, WC1E 6DD, United Kingdom; ∥ UCL School of Pharmacy, Faculty of Life Sciences, University College London, London, WC1N 1AX, United Kingdom; ⊥ School of Life Sciences, Pharmacy & Chemistry, Kingston University, Penrhyn Road, Kingston upon Thames, KT1 2EE, United Kingdom

**Keywords:** X-ray, Biomaterials, Dark-Field, Contrast Agents, Nanoparticles

## Abstract

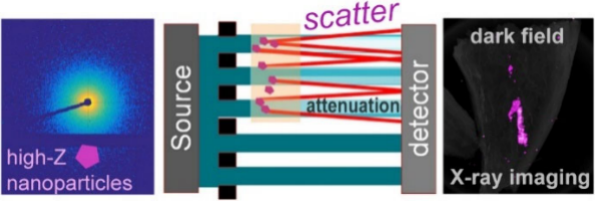

The poor soft tissue contrast of X-ray CT necessitates contrast agent use to improve diagnosis across disease applications, yet their poor detection sensitivity requires high injected doses, which restrict use in at-risk populations. Dark-field X-ray imaging is emerging as a more sensitive alternative to traditional attenuation-based imaging, leveraging scattered radiation to produce contrast. Yet aside from large, short-lived microbubbles, the alternate physics of dark-field detection has yet to be exploited for contrast agent development. Here we demonstrate that high-Z nanoparticles can provide a new means to producing dark-field image contrast, promoting scatter via a higher rather than lower electron density compared to microbubbles, increasing detection sensitivity compared to attenuation-based detection of a clinical iodine-based agent at an equivalent X-ray dose. As the use of dark-field X-ray imaging expands into more common clinical usage, this will support the development of a new class of nanoparticulate contrast agents.

Contrast agents are commonly used diagnostic aids, enhancing the radiological visibility and quantification of specific physiological structures, functional processes,^1^ or molecular targets associated with pathology.^2−4^ Beyond this, applications in tracking implanted biomaterials,^5−7^ gene expression,^8^ cellular therapies,^9^ and drug delivery^10^ are emerging to inform basic research and its translation. Yet despite traditional absorbance-based X-ray imaging being the most established, routinely used, and cost-effective clinical imaging modality, it suffers from poor sensitivity to contrast agents compared to alternatives such as MRI and nuclear imaging.^11^ This requires relatively high concentrations of contrast agents to be administered to patients, leading to side effects in a small minority of patients, including immunogenic, renal, and thyroid toxicity. This limits their application, leading to calls for development of better and more sensitive methods of contrast production.^12^

Dark-field (DF) X-ray imaging offers a potential solution to this challenge, with orders of magnitude sensitivity gains vs traditional absorbance-based CT.^13^ However, as an emerging modality only beginning to see clinical use,^14,15^ the development of contrast agents exploiting the alternative contrast mechanisms of dark-field imaging is currently underexplored. Like dark-field optical microscopy, its X-ray imaging counterpart gathers signal only from scattered photons, with directly transmitted light excluded from the image, thus reducing background signal. Though multiple hardware geometries and retrieval processes have been implemented for producing dark-field images, the 2D beam tracking approach is particularly attractive due to its ability to simultaneously produce phase-contrast and attenuation-based images alongside the dark-field channel, thereby maximizing signal usage (Figure 1A,B).

**Figure 1:**
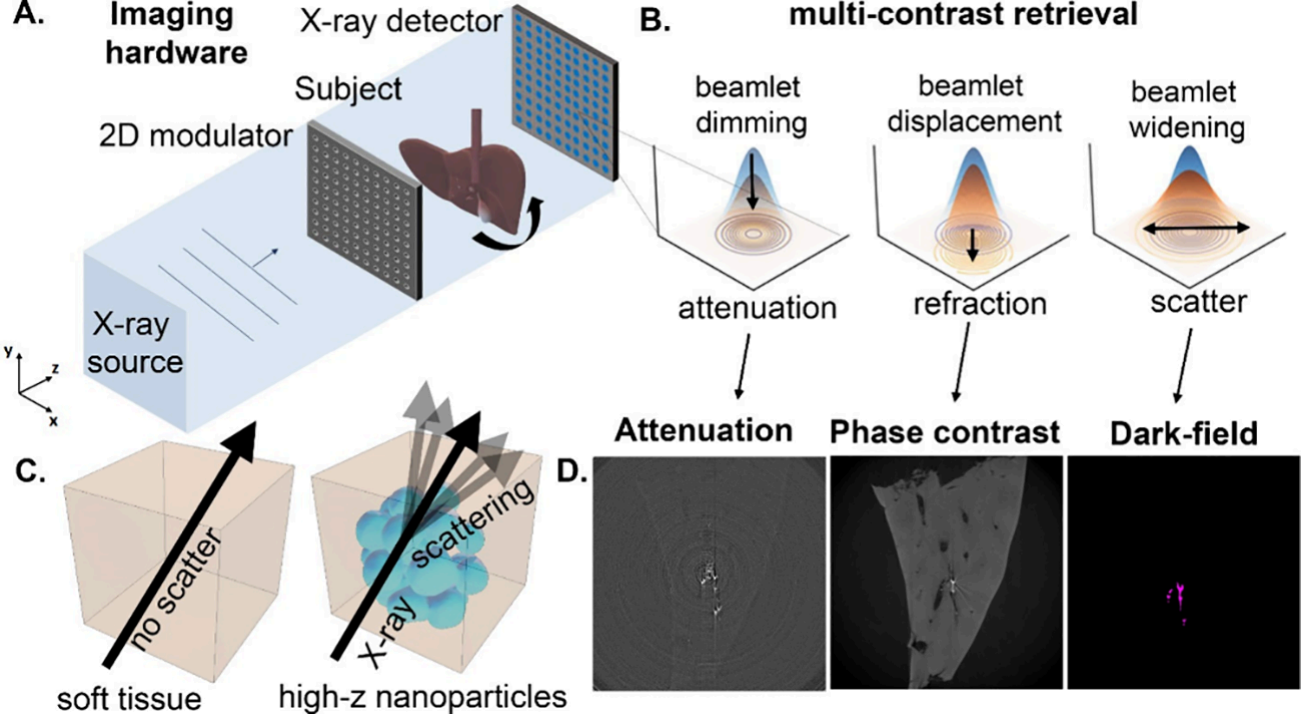
Schematic illustrating the beam tracking approach used here for multicontrast X-ray imaging including (A) hardware and rotating sample setup for 2D beam tracking CT and (B) corresponding beamlet profiles from which attenuation, phase contrast, and dark-field contrast were retrieved, respectively. Note that although these are not drawn for simplicity, the sample in (A) is illuminated by an array of equally spaced pencil beams. (C) Contrast mechanism showing X-ray scattering at the electron density interface between the surrounding tissue and high-Z nanoparticles. (D) Example attenuation, phase, and dark-field images of a mouse liver with only the injected nanoparticle contrast agent and not the soft tissue visible in the dark-field channel.

As X-ray scattering occurs predominantly at interfaces between materials with large differences in electron density,^16^ these features in particular should be exploited in the development of novel dark-field contrast agents. More specifically, the range of scattering angles best suited to dark-field imaging is encompassed within the small and ultrasmall angle X-ray scattering (SAXS and USAXS) windows,^17,18^ which occurs most efficiently at density variations between structures at the nanoscale to low microscale^16^ (Figure 1C). Exploiting this phenomenon, gas-filled microbubbles have been proposed and demonstrated as effective contrast agents for scatter-based X-ray imaging, whereby their lower internal electron density versus surrounding blood or tissue provides the scattering interface.^19−24^ Despite the advantage of also being a safe and clinically available contrast agent for ultrasound imaging, microbubbles also carry several disadvantages including poor shelf life and short half-life *in vivo* (1–7 min),^25^ while their size (1–10 μm diameter) is likely to prevent use in applications requiring extravasation. Though coating of microbubbles with high-Z nanoparticles including iron has been previously assumed to have no effect on scattering,^22^ iron oxide (magnetite) pigments found in acrylic paint have been implicated in the production of dark-field contrast,^26^ while attachment of gold colloids to microbubble contrast agents has been shown to provide an enhancement of dark-field signal.^27^ More recently, larger iron oxide microstructures (0.94–1.4 μm) have also been shown to produce dark-field X-ray contrast in a gene delivery context, at a relatively high concentration of 25 mg/mL.^28^ Despite these results, the evaluation of nanoparticle contrast agents alone remains an unexplored avenue for scatter-based signal generation.

To address this, we evaluated a series of high-Z nanoparticle materials for their ability to generate dark-field contrast, showing good detectability at low concentrations (0.5 mg/mL) in tissue-simulating phantoms and *exvivo* liver tissue (Figure 1C,D).

Four candidate nanoparticle types were chosen for their high-Z composition and commercial availability and characterized using dynamic light scattering (DLS) and scanning electron microscopy (SEM): see Table 1 and Figures 2A–C and S1–S5. Powder X-ray diffraction and energy dispersive X-ray spectroscopy (EDS) gave expected patterns for each material, respectively, confirming their reported compositions (Figures S6–S9).

**Table 1:** Summary of the Physical Parameters of the Four Nanoparticle Types Used Throughout This Study

**Material**	**Size (nominal)**	**Size (DLS)**	**Size (SEM) ± SD**	**Zeta potential (mV)**	**Catalogue code**
Platinum (Pt)	50	119 ± 5 nm	48 ± 13 nm	–8.2	685453
Platinum (Pt)	200	155 ± 30 nm 420 ± 24 nm	238 ± 89 nm	+2.4	771937
Barium titanate (BaTiO_3_)	<100 nm	n/a	50 ± 10 nm	–2.3	467634
Titanium dioxide (TiO_2_)	<100	151 ± 5 nm	103 ± 26 nm	+29.2	634662

**Figure 2:**
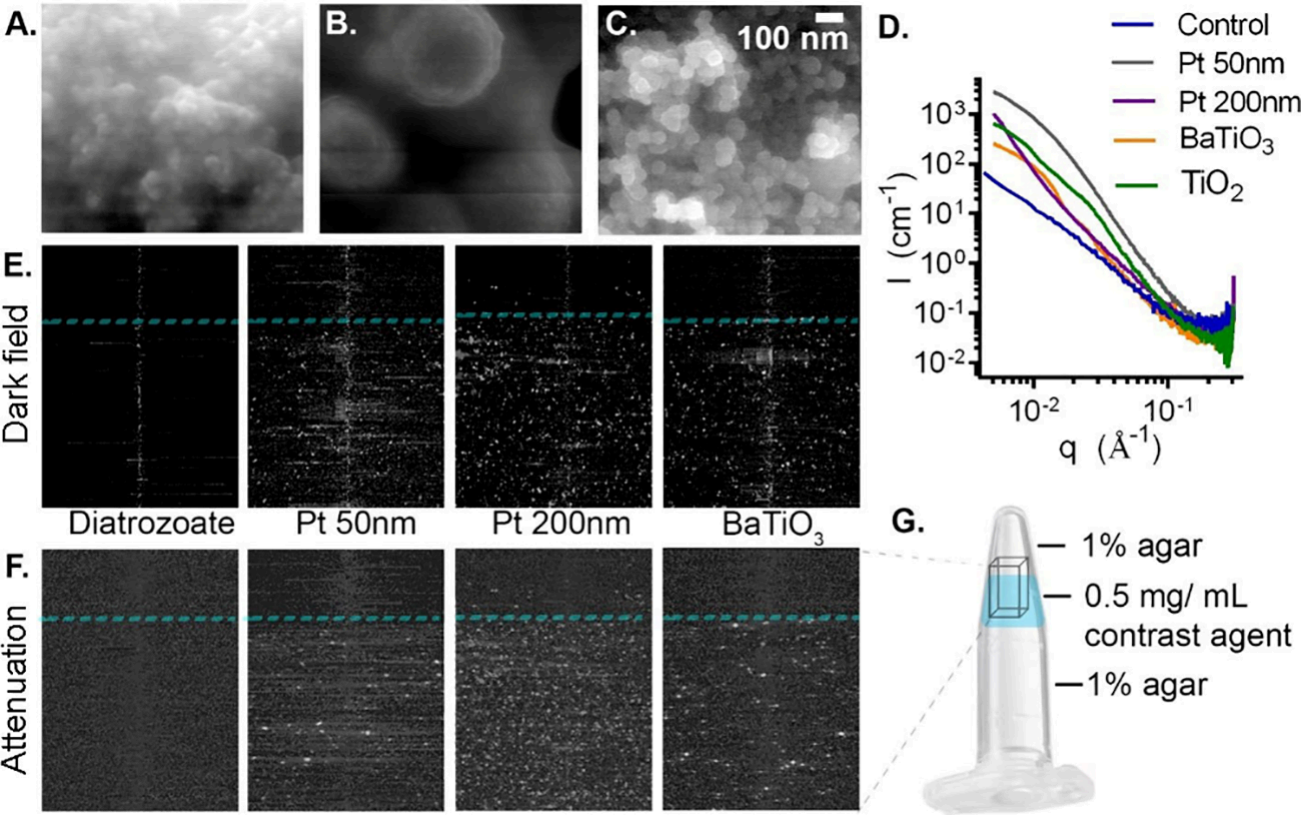
Representative scanning electron micrographs of platinum nanospheres at (A) 50 nm and (B) 200 nm and (C) barium titanate nanoparticles (<100 nm). Scale bar in C is representative for all SEM images. (D) Small-angle X-ray scattering (SAXS) was quantified in 1% agar for 50 and 200 nm platinum nanospheres, BaTiO_3_ nanoparticles (<100 nm), and TiO_2_ (<100 nm) at a concentration of 10 mg/mL, showing increased scattering vs 1% agar alone (control). (E) Dark-field and (F) attenuation CT maximum intensity projections for the volume indicated in G, showing increased signal in the dark-field and attenuation channels in the presence of nanoparticles (0.5 mg/mL) compared to agar alone. Diatrizoate showed no contrast in either attenuation or dark-field channels at the same concentration compared with agar alone. (G) Schematic of the phantom and region of interest location for X-ray tomography acquisition using a 1D mask.

To evaluate their suitability for dark-field X-ray imaging, small-angle X-ray scattering (SAXS) was quantified over a range of angles for each nanoparticle type suspended at 10 mg/mL in simulated tissue (1% agar), using an agar-only sample as a negative control (Figure 2D, Figures S10–S14). Scattering was elevated in all four nanoparticle samples compared to the control, with the 50 nm platinum nanospheres producing the highest scattering over the measured angle range, followed by 200 nm nanospheres of the same material and barium titanate(IV) nanoparticles (<100 nm). Lack of well-defined form factor oscillations was consistent with their relative polydispersity, as measured by DLS (Figure S1).

## High-Z Nanoparticles Are Detectable with Dark-Field X-ray Imaging Using 1D Beam Tracking

To evaluate their ability to produce dark-field X-ray contrast, tissue-simulating phantoms were produced with 0.5 mg/mL nanoparticle suspensions in 1% agar, with an agar-only layer as an internal control (Figure 2G). The phantoms were imaged at Diamond Light Source beamline I13-2 with a mean energy of 27 keV from a filtered pink beam. A 1D beam tracking approach (Figure S15), as described previously,^13^ was used to simultaneously acquire attenuation and scattering (dark-field) projection images from selected samples and reconstruct them into 3D tomographic volumes with 9 × 9 × 10.4 μm^3^ voxel size. These were visualized as maximum intensity projections, giving clear attenuation and dark-field contrast for each of the three initially tested nanoparticle types (Figure 2E,F). Similar images were also acquired for copper nanoparticles showing wider applicability (60–80 nm; Figure S16). For comparison, a current clinical iodine-based contrast agent (diatrozoate, also known as Hypaque or Gastrografin^(R)^) was imaged under the same conditions, giving no signal above background in either attenuation or dark-field channels (Figure 2E,F). This was quantified by calculating the contrast to noise ratio (CNR), giving a CNR_DF_ of 0.0886 and CNR_At_ of 0.0099 for diatrozoate in the dark-field and attenuation channels respectively. The lack of dark-field contrast here was consistent with the absence of material interfaces necessary for X-ray scattering in soluble small-molecule iodinated contrast agents (as opposed to high-Z nanomaterials), and the lack of attenuation contrast was consistent with its typical use at much higher concentration (250–370 mg/mL) in patients.^29^ For the nanoparticle samples, a trend of similar but slightly higher CNR was found in the dark-field compared to the attenuation channel, with values of CNR_DF_ = 1.94 and CNR_At_ = 1.63 for Pt50 nm, CNR_DF_ = 2.50 and CNR_At_ = 2.08 for Pt200 nm, and of CNR_DF_ = 2.12 and CNR_At_ = 1.53 for BaTiO_3_.

## 2D Beam Tracking Approach for High-Resolution Imaging

**Figure 3:**
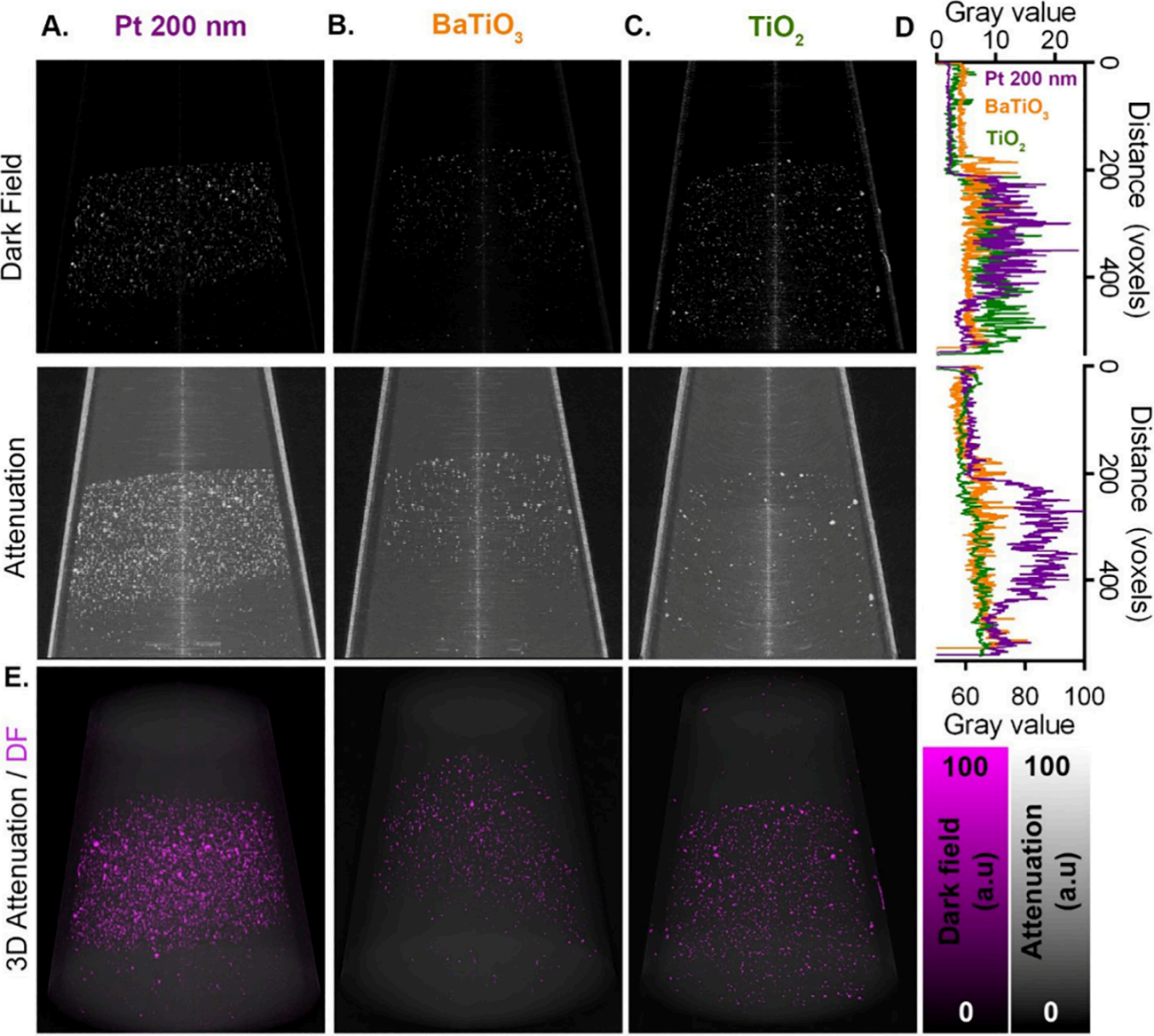
Transverse sections showing maximum intensity projections (100 slices) in attenuation and dark-field (*y* axis) channels of layered 1% agar alone and with 0.5 mg/mL (A) 200 nm platinum nanospheres, (B) BaTiO_3_ nanoparticles (<100 nm), and (C) TiO_2_ nanoparticles (<100 nm). (D) Line profile of mean dark-field and attenuation signal intensity across the section averaging 150 voxels. (E) 3D volume renders of overlaid attenuation and dark-field channels for each sample.

To further evaluate nanoparticle performance in dark-field imaging, selected samples were imaged with a 2D beam tracking approach^30^ (Figure1A,B). This allows for isotropic resolution imaging compared to the 1D tracking method used above, though at the expense of reduced photon flux due to the smaller grating open fraction. The mean energy of the beam was also 27 keV, and the voxel size in the reconstructions was 10 × 10 × 10 μm^3^. Attenuation, phase, and *x* and *y* directional scattering (dark-field) images were retrieved for each sample, showing elevated contrast in all channels for the nanoparticle-based agents at 0.5 mg/mL compared to control (agar only) regions (Figure 3A–E, Figures S17, S18). This was again confirmed with elevated CNR being found in the dark-field versus attenuation channel (Table S2). Visualization as both 2D slices (Figure 3A–C) and 3D volumes (Figure 3E) showed clear separation of contrast-agent-containing and background agar-only regions in dark-field reconstructions, supporting the potential for each nanoparticle type to be used as a dark-field contrast agent. Line profile plots showed an increase in signal above the background noise for each sample, confirming that concentrations as low as 0.5 mg/mL were sufficient to enable detection (Figure 3D). Notably, while attenuation-based imaging showed contrast between the tissue-simulating agar and surrounding air outside the tube, the simulated tissue showed no contrast on dark-field images, demonstrating its ability to reduce soft-tissue background signal in favor of dark-field specific scattering agents (Figure 3A–C). In addition to visibility on maximum intensity projections, individual points of hyperintensity were also visible on single slices (10 μm isotropic voxel resolution) in nanoparticle-containing but not agar-only regions, demonstrating the resolution of the technique. Histograms for dark field (*y*-axis) gray values of each pixel were plotted over 100 slice regions of interest for background (agar only) regions and the equivalent nanoparticle-containing regions for each sample (Figure S19), confirming their increased numbers of high-intensity pixels.

To rule out confounding sources of dark-field contrast such as air bubbles (which may also appear hyperintense due to their nano- or microscale electron density boundaries), a voxel-wise correlation analysis was then performed between dark-field and attenuation channels, where nanoparticles should appear hyperintense in both. Consistent with the presence of nanoparticles as the main source of dark-field contrast, a positive spatial correlation was found between signal in attenuation and dark-field channels. A Pearson’s correlation of 0.64 (*x*) and 0.95 (*y*) was found for 200 nm platinum, while lower correlations were found for the lower-*z* particles, consistent with their weaker signal in the attenuation channel, with 0.35 (*x*) and 0.71 (*y*) for BaTiO_3_ and 0.36 (*x*) and 0.45 (*y*) for TiO_2_ (respectively attenuation versus scatter *x*, and vs scatter *y*). No correlation between channels was found in the agar-only control region: −0.07 (*x*) and −0.03 (*y*).

## Detection in a Biological Sample

**Figure 4:**
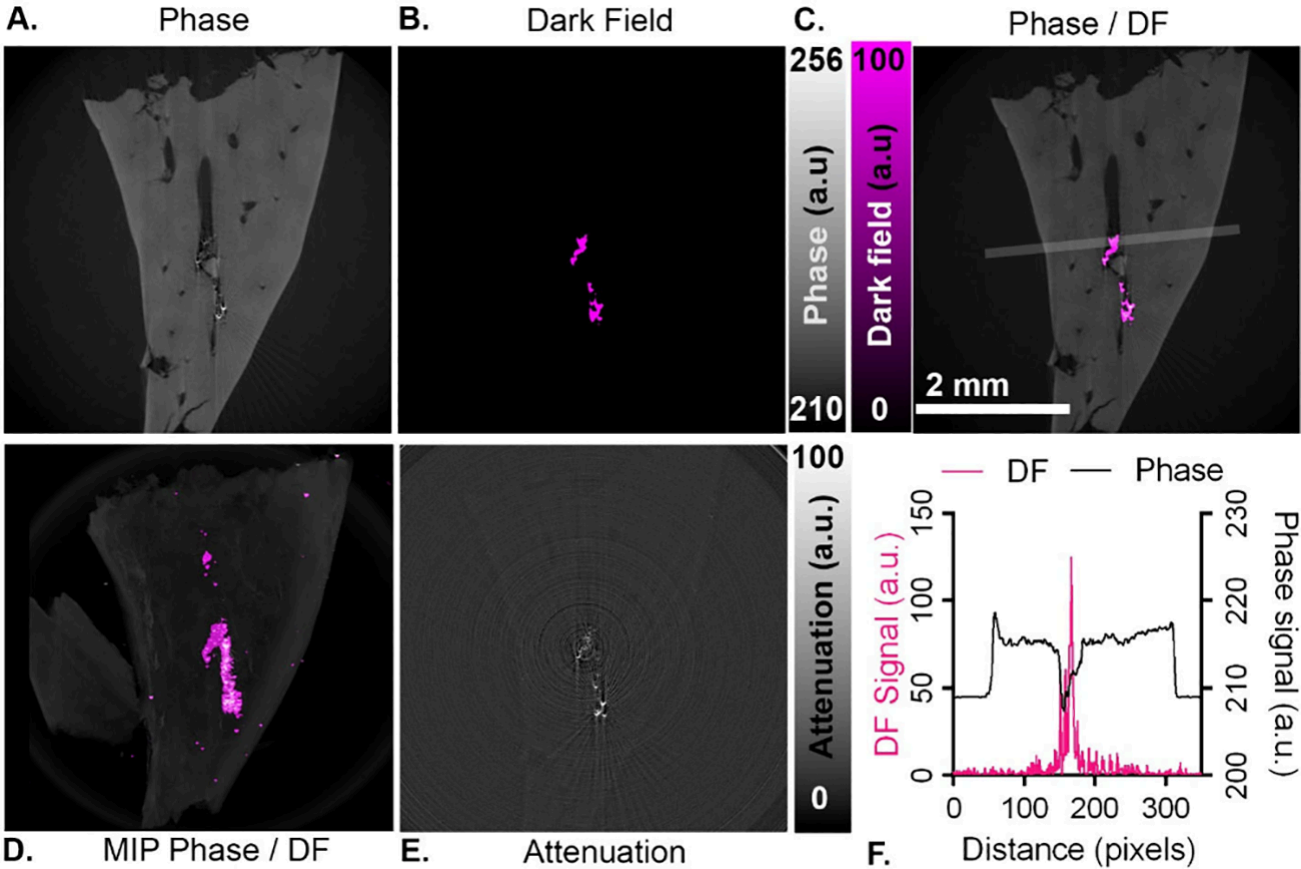
Mouse liver lobe injected along the hepatic vein with a 10 mg/mL suspension of platinum nanoparticles (200 nm), shown as single reconstructed CT slices (7.5 μm in-plane pixel size) in (A) phase, (B) dark field (*y*-axis), and (C) overlaid phase and DF planes. (D) 3D maximum intensity projection of the whole liver sample shows both phase and dark-field channels. (E) Attenuation slice showing the same plane as in parts A–C. (F) Profile plot for dark-field (DF) and phase channels across a 15-pixel wide area indicated in C, showing colocalization of the dark-field signal intensity with the lower intensity phase signal of the venous lumen.

To evaluate the ability to distinguish nanoparticle-derived dark-field contrast from physiological structures, a 10 mg/mL suspension of Pt nanoparticles (200 nm) was injected along the hepatic vein of a lobe of mouse liver and imaged using the 2D beam tracking approach as described above. The tissue was imaged with a 27 keV [pink] beam, and the reconstructed volumes had a voxel size of 6.25 × 6.25 × 6.25 μm^3^. Retrieval of phase contrast images showed good soft tissue contrast (Figure 4A), including the hepatic vein lumen and bile ducts, neither of which could be easily distinguished in the attenuation image (Figure 4E). Clear evidence of signal was seen on the dark-field channel at the site of injection (Figure 4B–F), which was confirmed as the hepatic vein using phase contrast for anatomical reference and a line profile plot in addition to an overlaid image to visualize spatial colocalization (Figure 4C,F). This also gave a clear indication of the low background signal generated by soft tissue in the dark-field channel (in contrast to attenuation and phase-contrast images), highlighting its specificity for nanoparticle contrast agent detection. Comparison of CNR between the injection site and background liver tissue again showed an increased value for the dark-field channel compared to attenuation (45.98 CNR_DF_ vs 25.33 CNR_At_).

## Concluding Remarks

Here we present the concept of using high-Z nanoparticles as contrast agents for dark-field X-ray imaging, demonstrating experimental feasibility with nanomaterials ranging in atomic number from ^78^Pt and ^58^Ba down to ^29^Cu and ^22^Ti and between 50 and 200 nm in diameter. Going beyond previous work in which microbubbles were shown to act as effective DF contrast agents,^21−24^ here we took the opposite approach to enhancing X-ray scatter: exploiting low- to high-density interfaces between the typical aqueous physiological environment and the relatively denser core of metal-based nanoparticles. Though this study was limited in its use of unfunctionalized/uncoated nanoparticles, various established methods exist to coat these for improved aqueous stability and *in vivo* injection.^31,32^ Indeed, interest already exists in the use of BaTiO_3_ and TiO_2_ as agents for drug delivery, photothermal and piezoelectric therapy,^33,34^ and antimicrobial and regenerative medicine applications,^35−37^ pointing toward combined theranostic potential.

Encouraging results were obtained in tissue-simulating phantoms as well as in *ex vivo* liver tissue. Nanoparticles were found to be detectable at concentrations of 0.5 mg/mL, at which point a commonly used clinical iodine-based contrast agent was undetectable in attenuation-based images acquired with the same X-ray dose (Figure 2). In liver tissue, dark-field contrast was shown to be unaffected by soft tissue structure, which was visible in only the attenuation and phase-contrast channels. As the separation of signal originating from contrast media versus native tissue can be a challenge in conventional CT due to the variability of endogenous background attenuation,^3^ the presence of only nanoparticle-based signal in the dark-field channel provides a means to remove this ambiguity.

Over 300 million CT scans are performed per year,^12^ and with each increasing patients’ cancer risk in a radiation dose-dependent manner, methods to acquire diagnostic information with reduced X-ray exposure have the potential to reduce cancer incidence at a global level.^38^ With clinical DF-CT devices now emerging and claimed to reduce X-ray dose 100-fold compared to traditional scans,^15^ the development of compatible contrast agents should be a priority for future work in this field.

Though a number of physical and computational approaches have been demonstrated for producing dark-field X-ray images, we focus here on the use of the single grating beam tracking method for its effective exploitation of the dose delivered to the subject, enabling simultaneous acquisition of attenuation and phase-contrast signal.^13,15,39,40^ This allows images with multiple and complementary modes of contrast to be obtained with a single scan, for example, providing physiological data such as liver architecture in a distinct channel from that of the contrast agent (see Figure 3). Though our setup used pixel sizes in the low-micron range, no loss of dark-field sensitivity has previously been found at pixel sizes up to 2 orders of magnitude higher than those used here from both synchrotron^41^ and standard X-ray tube-based sources,^42^ supporting this technique’s scalability to clinical dimensions. Indeed compared to the system used here, the larger pixel sizes used in clinical scale systems help compensate for their lower photon flux vs synchrotron sources, allowing signal collection over orders of magnitude greater pixel area, ensuring DF imaging feasible even at clinically appropriate X-ray doses.^43^

As various interacting factors are known to affect small-angle scattering, including X-ray voltage, material composition, size, nanotopology (including surface roughness), and aggregation,^16^ future work is required to go beyond this proof-of-concept study to systematically screen candidate nanomaterials to optimize scatter and therefore signal generation. Though dark-field contrast generation from simple monodisperse SiO_2_ microspheres has previously been shown to be predictable with mathematical modeling,^44^ further work to take into account the many interacting factors discussed above has yet to provide a feasible alternative to experimental measurements. Future work too must consider nanoparticle biocompatibility and efficiency of signal generation within the beam parameters available on clinical dark-field imaging systems. For example, though our study used a beam energy of 27 kVp—at the lower end of the range used clinically^45^—voltages between 38 and 120 kVp are now being implemented in clinical tube-based DF systems.^14,15,23^ Future work should also explore sub-5 nm particles for their DF contrast, given that their renal clearance is enhanced,^46^ and small angle scattering is still known to occur at this size.^16^ Economic aspects of translation must also be taken into account in future studies, with more affordable high-Z materials such as barium likely to be more practical for scale-up to clinical doses than costlier platinum or gold agents. This is a problem that has already been explored to some degree in the development of high-Z nanoparticle contrast agents for traditional attenuation-based CT imaging, with a variety of existing materials already produced for *in vivo* use.^47,48^

## Supporting Information Available

Additional experimental details, materials, methods, and data (including DLS, SEM, XRD, EDS, SAXS, and reconstructed CT images) (PDF)
