# Supplementary material for: Nanoparticle Contrast Agents for Dark-Field X-ray Imaging
Source: Nano Lett. 2024 Nov 27;25(3):1036–42. doi: 10.1021/acs.nanolett.4c04878 (PMC11760164; doi:10.1021/acs.nanolett.4c04878)
Supplement: Supplementary file 1 — nl4c04878_si_001.pdf [file nl4c04878_si_001.pdf]

Electronic Supporting Information

to

Nanoparticle Contrast Agents for Dark-Field X-Ray Imaging

Carlos Navarrete-León[ab], Adam Doherty[ab], Margarita Strimaite[cd], Joseph C. Bear[e], Alessandro Olivo[a], Marco Endrizzi[ab], P. Stephen Patrick[c*]

a. Department of Medical Physics and Biomedical Engineering, University College London, London, WC1E 6BT, United Kingdom

b. X-ray microscopy and tomography lab, The Francis Crick Institute, London, NW1 1AT, United Kingdom

c. Centre for Advanced Biomedical Imaging, Division of Medicine, University College London, London, WC1E 6DD, United Kingdom

d. UCL School of Pharmacy, Faculty of Life Sciences, University College London, London, WC1N 1AX, United Kingdom

e. School of Life Sciences, Pharmacy & Chemistry, Kingston University, Penrhyn Road, Kingston upon Thames, KT1 2EE, United Kingdom

*peter.patrick@ucl.ac.uk

Supplementary Methods

Methods

Materials

All nanoparticles were obtained in powder form and used as supplied, including Platinum nanopowder (200nm by SEM; Sigma Aldrich Ltd.: 771937), Platinum nanopowder (50nm by TEM; 685453), Barium titanate nanopowder ($BaTiO_3$(IV) cubic crystalline; <100nm by BET; Sigma Aldrich: 467634), and Titanium oxide nanopowder (TiO_2 (IV), mix of anatase and rutile, <100nm by BET; Sigma Aldrich: 634662). Diatrozoic acid (Sigma Aldrich Ltd.: D9268) was used as a control.

SAXS quantifaction

Nanoparticles were suspended in 1% agar (Agar-agar, Millipore) at 10mg/mL in 1.5mm OD borosilicate glass capillary tubes (Capillary Tube Supplies Ltd, Bodmin, Cornwall) and small angle X-ray scattering (SAXS) measured using a dedicated SAXS device (Ganesha 300XL, SAXSLAB). A 1% agar only sample was used as a negative control. Signal was collected over 300s for all samples using a sealed tube Cu-Kα (λ = 1.54184 Å) X-ray source at 50kV. 1D and 2D scattering patterns were extracted using SAXSGUI (Rigaku Innovative Technologies Inc.).

Material Characterisation

Dynamic light scattering measurements were performed using a Zetasizer Ultra instrument and ZS Xplorer software (Malvern Panalytical). Stock suspensions of particles were prepared at a concentration of *ca*. 5 mg/ml, and diluted to a final concentration of *ca*. 0.04 mg/ml. Each measurement was performed in triplicate.

Powder X-ray diffraction patterns were collected using a Rigaku Miniflex 600 diffractometer (Cu Kα radiation, λ = 1.54184 Å) over the 2θ range 20-70°, at 40 kV and 15 mA.

Scanning electron microscope images were obtained using a JEOL JSM-6701F field emission scanning electron microscope. Samples were mounted on adhesive carbon tabs and sputter coated with gold prior to imaging. Sample size analysis was performed in ImageJ using the "measure" function (n=70 to 140).

Sample production for X-ray imaging

Tissue simulating phantoms containing nanoparticles, as described above (or diatrozoate) were layered in 1% agar in 0.5mL PCR tubes, at 0.5 mg/mL, or injected into formalin-fixed mouse liver at 10 mg/mL.

Imaging set-up

The samples were imaged at Diamond Light Source beamline I13-2. The experiments were performed at a mean energy of 27keV from a filtered pink beam with platinum mirror and filters of 1.34 mm pyrolytic graphite and 3.2 mm aluminum. The samples were placed *ca*. 221 m from the source, and the modulators were placed 15 cm upstream of the sample. The distance between the sample and the detector was 64 cm and 128 cm for the 1D and the 2D modulators, respectively.

The 1D modulator was manufactured by Microworks GmbH (Germany) by electroplating gold on a 200 μm thick silicon substrate. and the modulator has an aperture width of 9 μm with a period of 36 μm. The 2D modulator was fabricated with laser-ablation from a 100 μm-thick tungsten foil (Goodfellow) with a period of 50 μm. The apertures have a conical shape with diameters of 15 μm in the front and 30 μm in the back.

Signal was detected using a pco.edge 5.5 (pco., Excelitas Technologies) camera coupled to a scintillator-objective combination with an effective pixel size of 2.6 μm × 2.6 μm and a field of view of 6.6 cm × 5.6 cm.

Image acquisition

All samples were scanned by acquiring projections while rotating over 180° in a fly-scan fashion. This was repeated at different modulator sub-pitch displacements, which were analysed separately and the resulting images then stitched to obtain an image with higher sampling.

and the images were then stitched to obtain an image with higher sampling. The 1D modulators were raster scanned laterally along *x*. The 2D modulators were raster scanned both in *x* and *y* to achieve isotropic resolution. Flat and dark images were acquired at each modulator position, before and after rotating the sample.

The phantoms with platinum nanospheres at 50nm, and 200nm, and Barium titanate nanoparticles (<100nm) were initially imaged with the 1D modulator. For each modulator position, 960 projections were acquired with an exposure time of 0.15 s per projection. The modulator was raster-scanned in in 4 lateral steps of 9 μm in *x*, leading to a total exposure time of 960 × 4 × 0.15 s = 9.6 min for each phantom.

The phantoms with 200nm platinum nanospheres, $BaTiO_3$ nanoparticles (<100nm), and TiO_2 nanoparticles (<100nm) were later imaged with the 2D modulator. For each modulator position, 800 projections were acquired with an exposure time of 0.1 s per projection. The modulator was raster-scanned in 5 × 5 positions, by using 10 μm displacements both in *x* and *y*. The total exposure time was 800 × 5 × 5 × 0.1 s = 33.3 min for each phantom.

The mouse liver lobe injected with 10mg/mL solution of platinum nanoparticles (200nm) was also imaged with the 2D modulator. In this case, 1200 projections were acquired with 0.15 s exposure time per projection. The modulator was raster-scanned in 8 × 8 positions, with 6.25 μm displacements both in *x* and *y*. This led to a total exposure time of 1200 × 8 × 8 × 0.15 s = 3.2 h.

Data analysis and tomographic reconstruction

The transmission, refraction, and dark-field images were obtained by comparing the intensities with [$I_s(x, y)$] and without [$I_0(x, y)$] the sample in each beamlet.

For the 1D acquisitions, images were binned vertically by a factor of 4, and a line profile was extracted for each detector row over 5 adjacent beamlets (each beamlet spreads over 15 pixels) and fitting a 7-Gaussian + m*x + c profile to each. A sliding window was moved in 3 beamlets increments along the detector row, and repeated until the signal has been retrieved for all beamlets. The amplitude, centre, and variance for the 3 central Gaussians only were extracted from the fits, and the comparison between their values with and without the sample is used to generate the transmission, refraction and dark-field signals, respectively [1,2]. Dithered images were then stitched to form a single projection.

For the 2D acquisitions, it was obtained by selecting a window of 20 × 20 pixels around each beamlet with and without the sample. The transmission was calculated by the sum of intensities in the windows. The two refraction images, by measuring the displacements between the beamlets with a subpixel cross-correlation algorithm [3]. The dark-field signals, by calculating the second moment of the intensity distributions, which represents the variance [4]. The phase shift was obtained by integrating the two refraction images throw a Fourier space method [5].

After retrieving the signals, they were reconstructed in tomography from the projections taken at different angles using the filtered back projection (FBP) implementation of the Astra Toolbox [6].

Image Analysis

Voxelwise correlation analysis (Pearson's Correlation) was performed using the Coloc 2 plugin for Fiji (ImageJ) across 160 slices for each sample in the agar only (control) and 0.5 mg/mL nanoparticle concentration regions.

Contrast to noise ratios were calculated in Fiji, using the formula (Mean pixel intensity sample – mean pixel intensity background)/(noise; measured as standard deviation in pixel intensity). For the 1D beam tracking acquisitions a 250x250 pixel central region through whole sample was rendered as a 3D maximum intensity projection (as shown in figure 2), and two 100x100 pixel regions of interest were drawn either side of the central axis in both background and particle containing areas for analysis. For the 2D beam tracking samples, a central 100 pixel slice was rendered as a maximum intensity projection (as shown in figure 3) for both dark field and attenuation channels, and 120x120 pixel regions of interest were drawn either side of the central axis in both background and sample containing regions for analysis. For the liver, particle containing regions were manually segmented in each channel on a slice-by-slice basis, and compared to a 200x200 pixel region of background liver tissue.

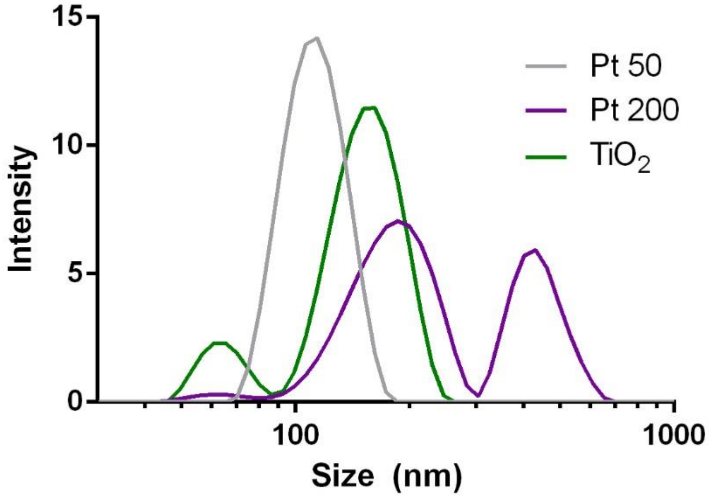

Figure S1. Dynamic light scattering (DLS) measurements showing particle size distribution (by scattering intensity) for three of the four candidate nanoparticles.

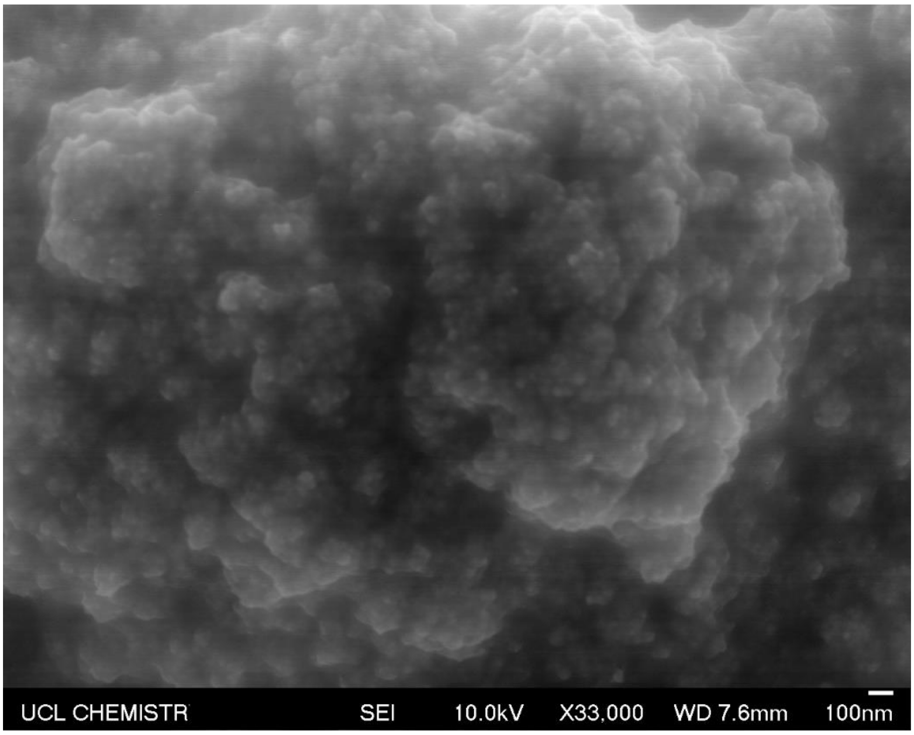

Figure S2. Scanning electron micrograph showing Pt 50 nanoparticles.

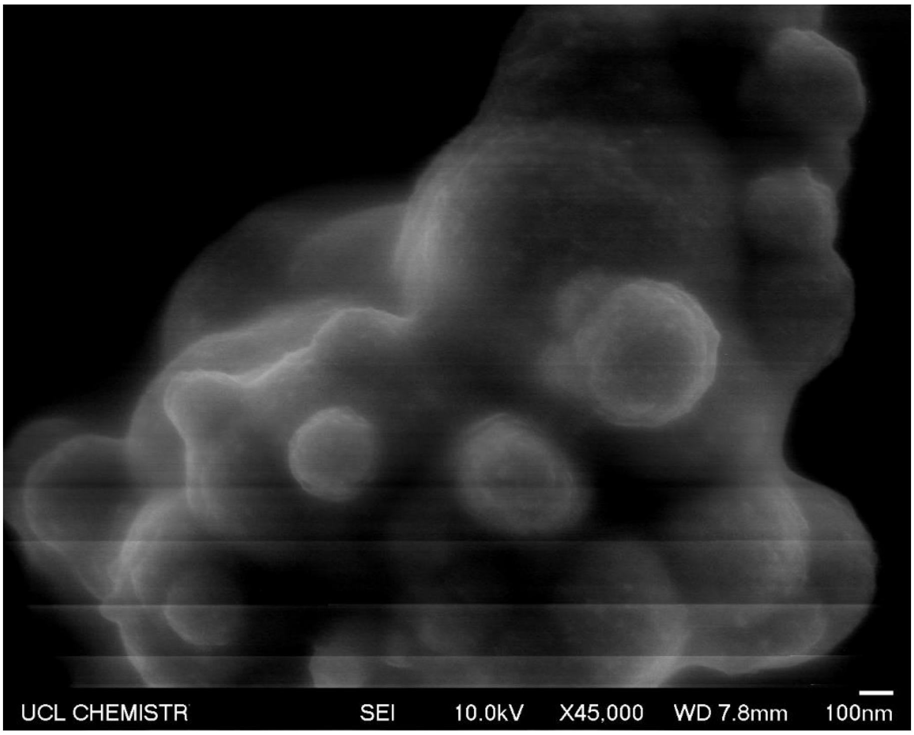

Figure S3. Scanning electron micrograph showing Pt 200 nanoparticles.

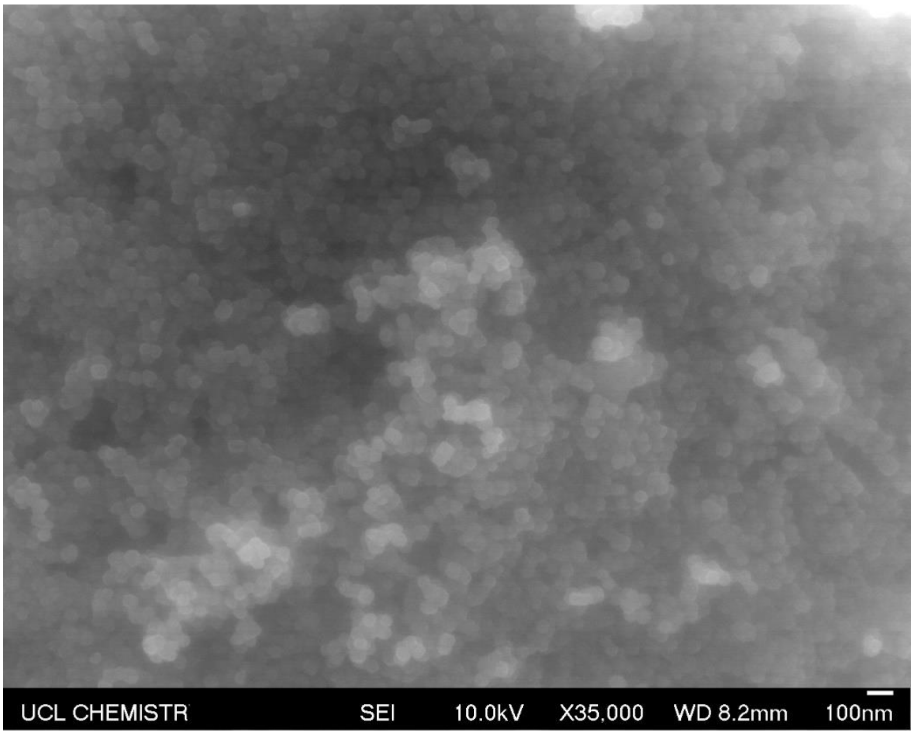

Figure S4. Scanning electron micrograph showing Barium titanate ($BaTiO_3$) nanoparticles.

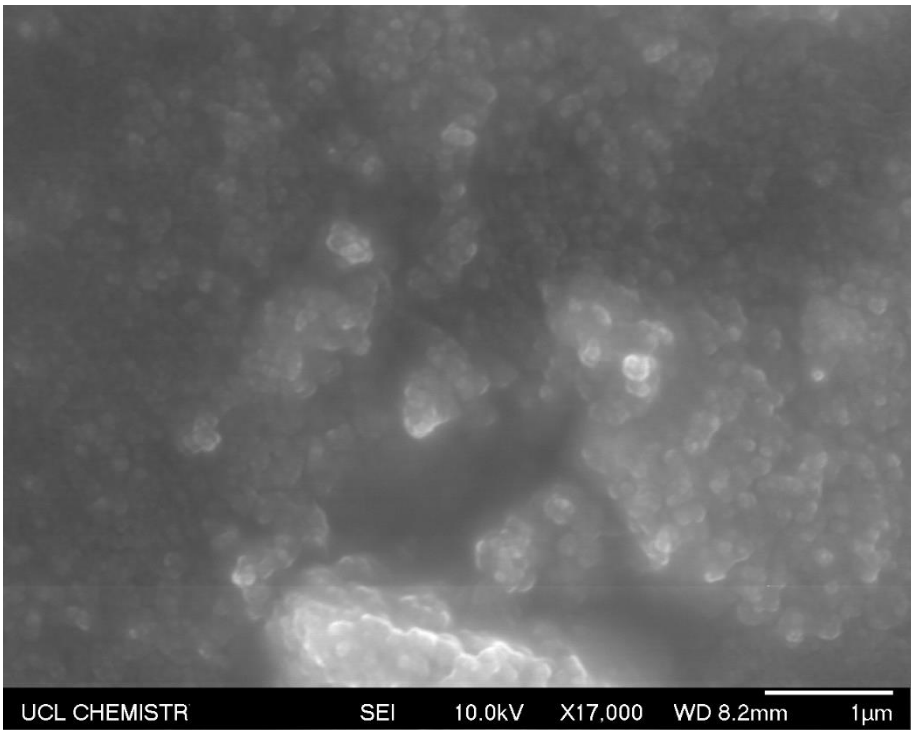

Figure S5. Scanning electron micrograph showing Titanium dioxide (TiO_2) nanoparticles.

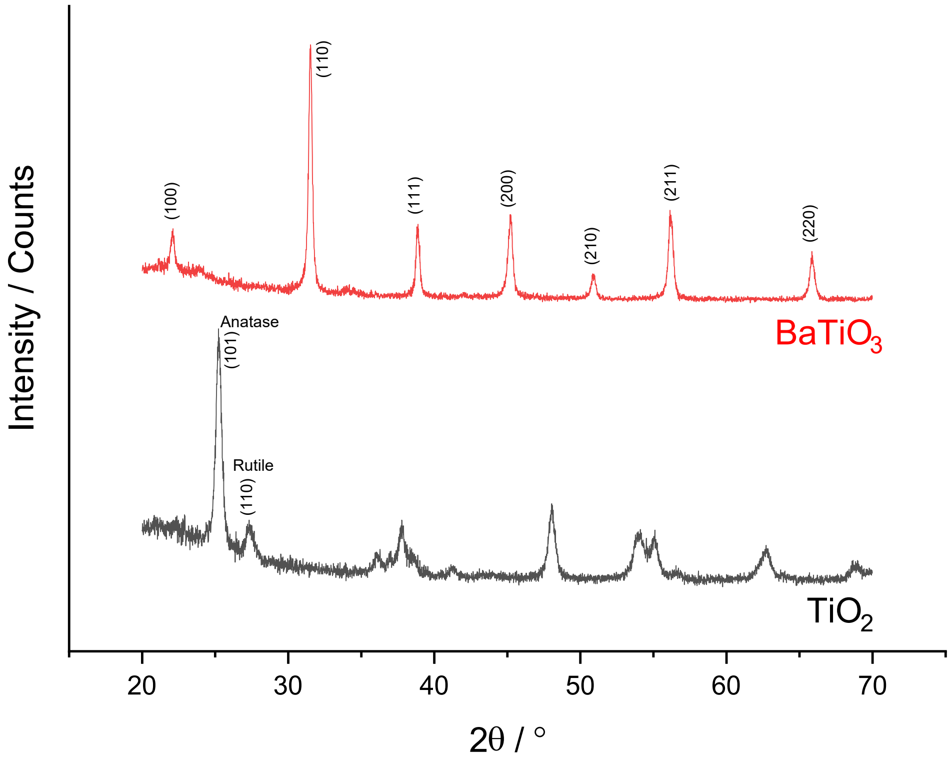

Figure S6. Powder X-ray diffraction patterns of $BaTiO_3$ and TiO_2 nanoparticles confirm the correct phases are present. Patterns were indexed using ICSD deposition numbers 67518 and 9852 respectively.

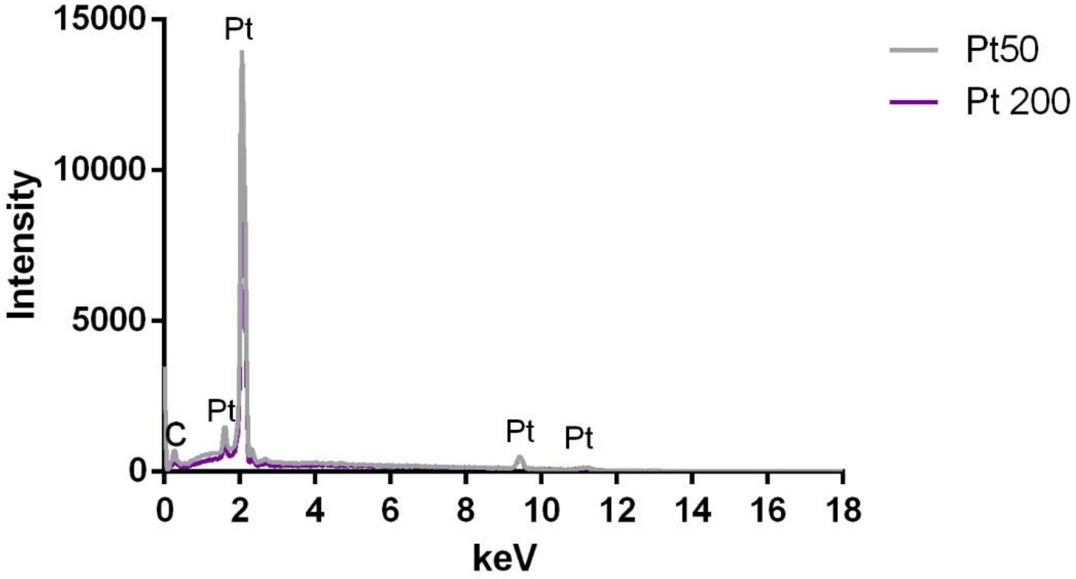

Figure S7. Energy dispsersive X-ray spectrum of platinum nanopowder (50nm and 200nm), showing the presence of platinum.

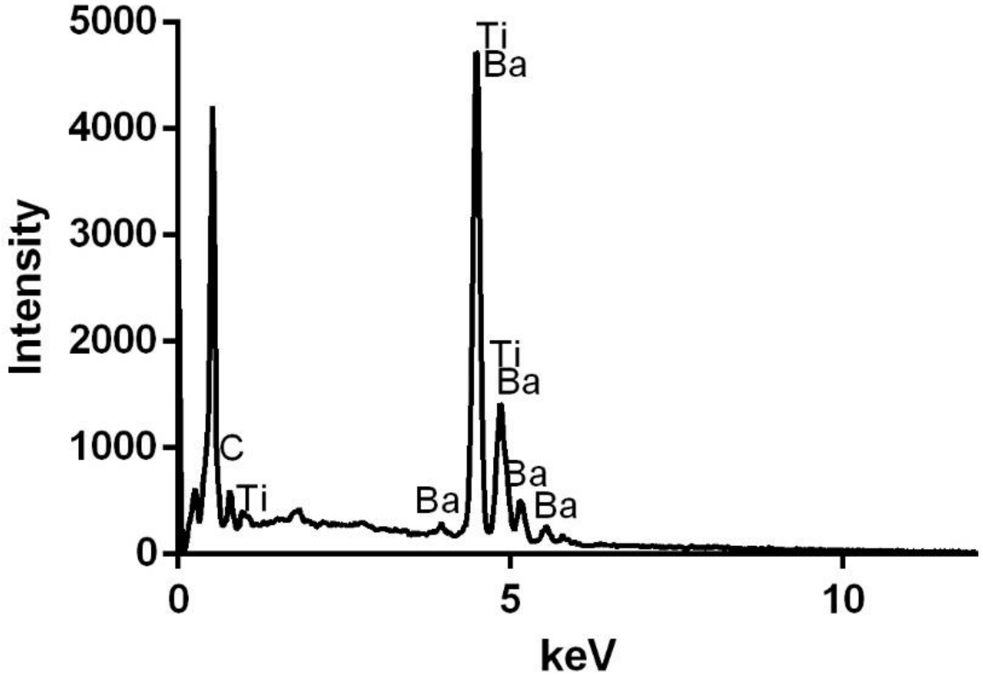

Figure S8. Energy dispersive X-ray spectrum of $BaTiO_3$ nanoparticles.

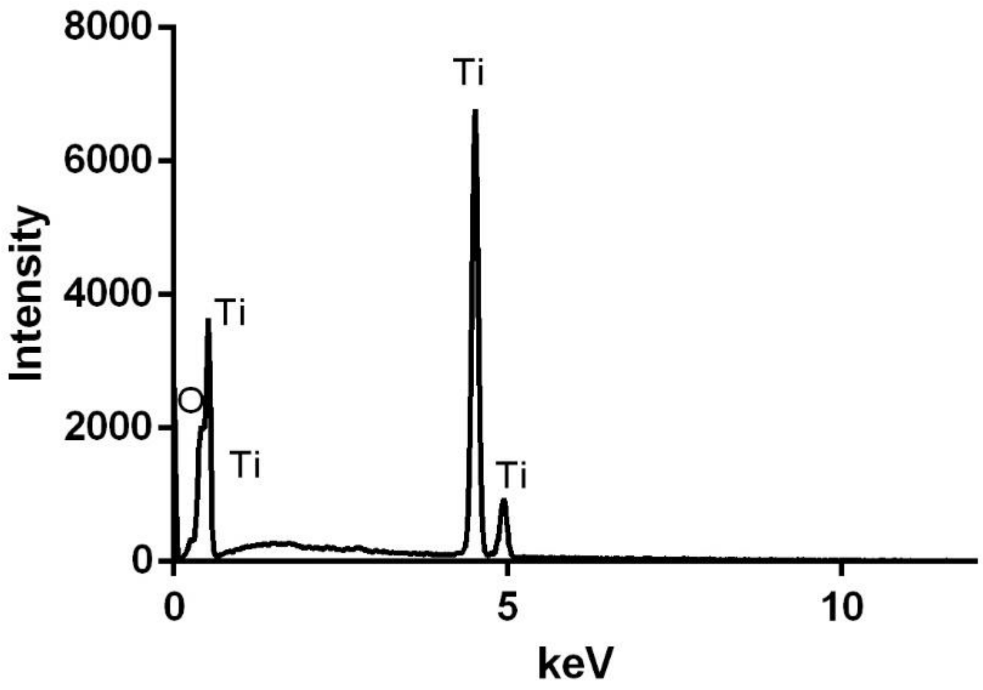

Figure S9. Energy dispersive X-ray spectrum of TiO_2 nanoparticles.

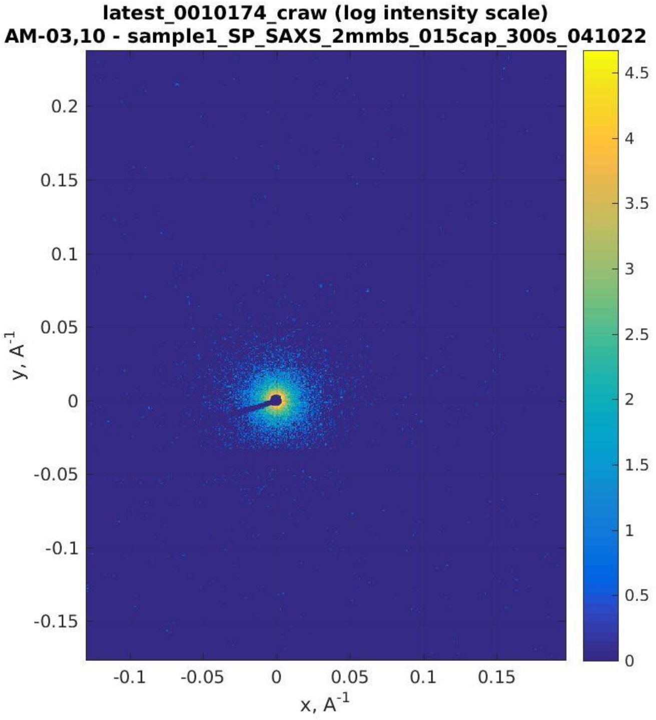

Figure S10. 2D SAXS pattern of 1% agar (negative control) acquired for 300s.

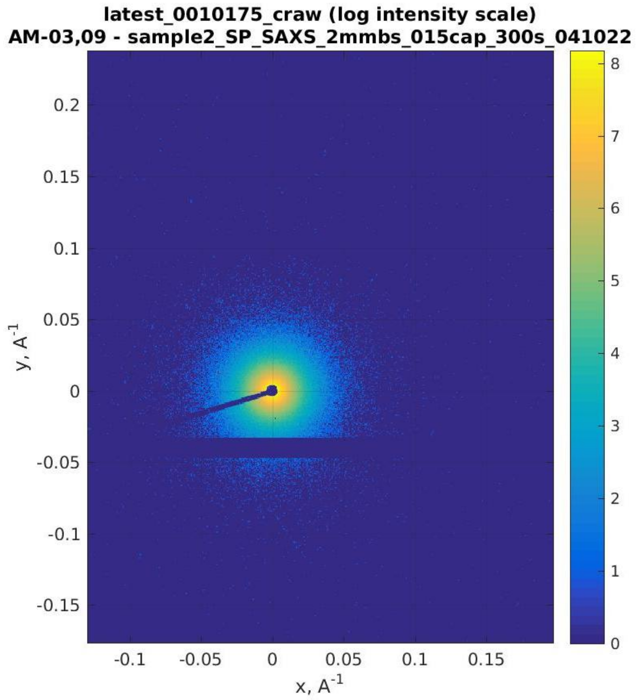

Figure S11. 2D SAXS pattern of 50nm Platinum nanospheres (10 mg/mL) in 1% agar (negative control) acquired for 300s.

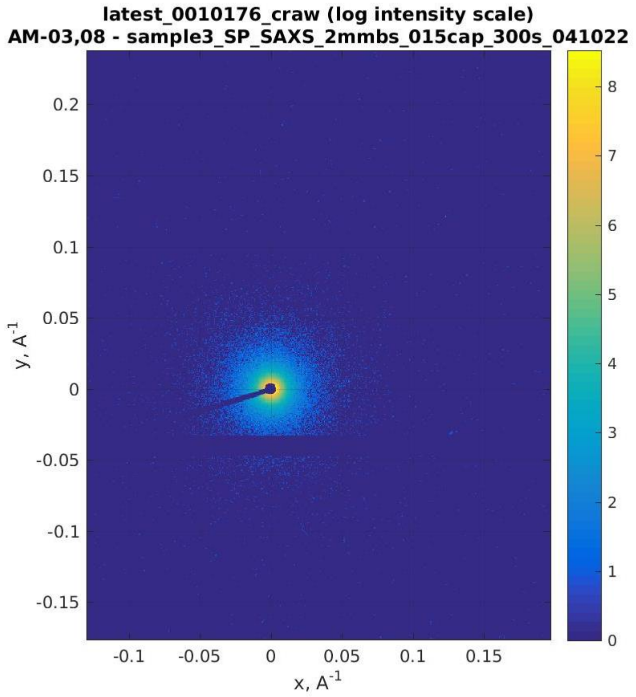

Figure S12. 2D SAXS pattern of 200nm Platinum nanospheres (10 mg/mL) in 1% agar, acquired for 300s.

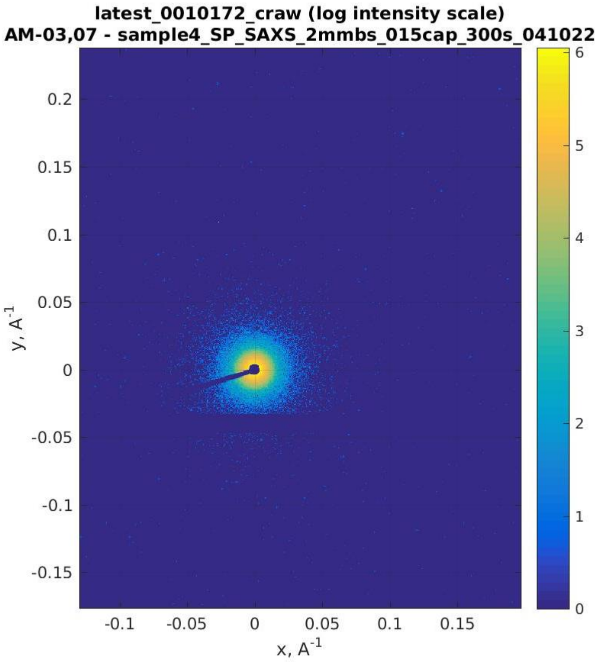

Figure S13. 2D SAXS pattern of $BaTiO_3$ nanoparticles (<100nm; 10 mg/mL) in 1% agar, acquired for 300s.

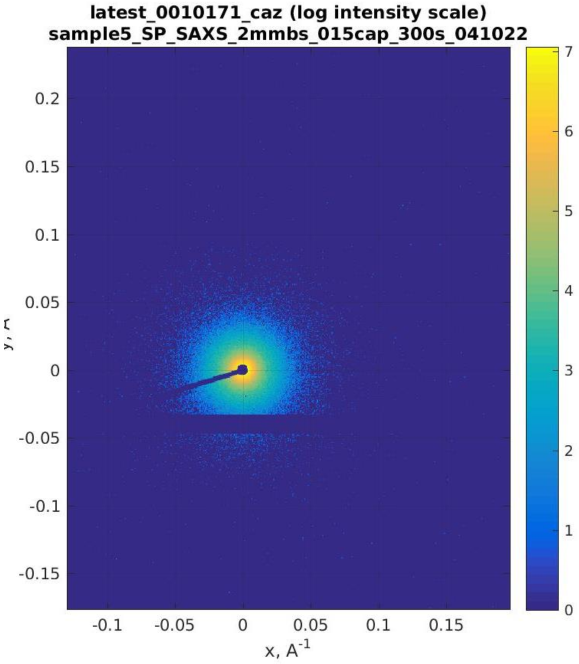

Figure S14. 2D SAXS pattern of TIO_2 nanoparticles (<100nm; 10 mg/mL) in 1% agar, acquired for 300s.

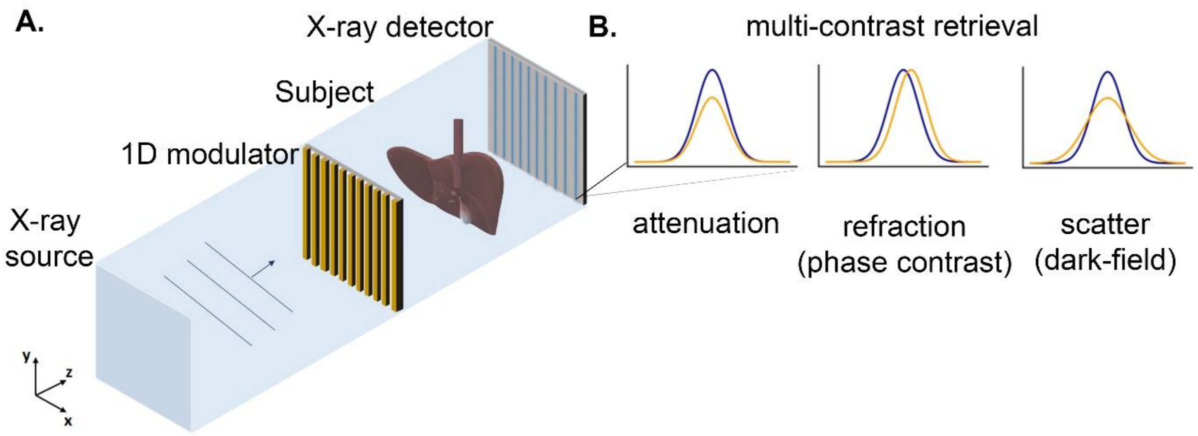

Figure S15 Schematic illustrating the beam tracking approach used here for multi-contrast X-ray imaging including **A.** hardware and rotating sample set for the 1D beam tracking approach, and **B.** corresponding beamlet profile analysis producing attenuation, phase contrast, and dark field retrievals respectively.

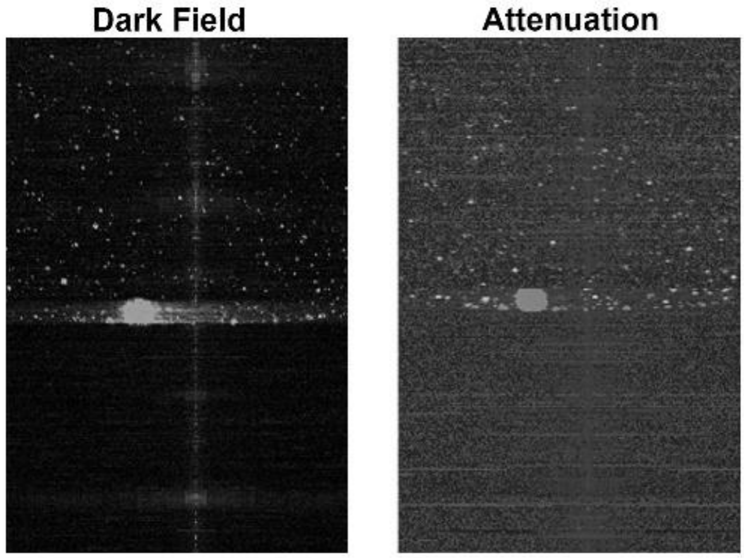

Figure S16. Maximum intensity projections of dark field (left) and attenuation (right) reconstructions of 0.5mg/mL copper nanoparticles (60-80nm) in 1% agar (top), layered on 1% agar alone (bottom).

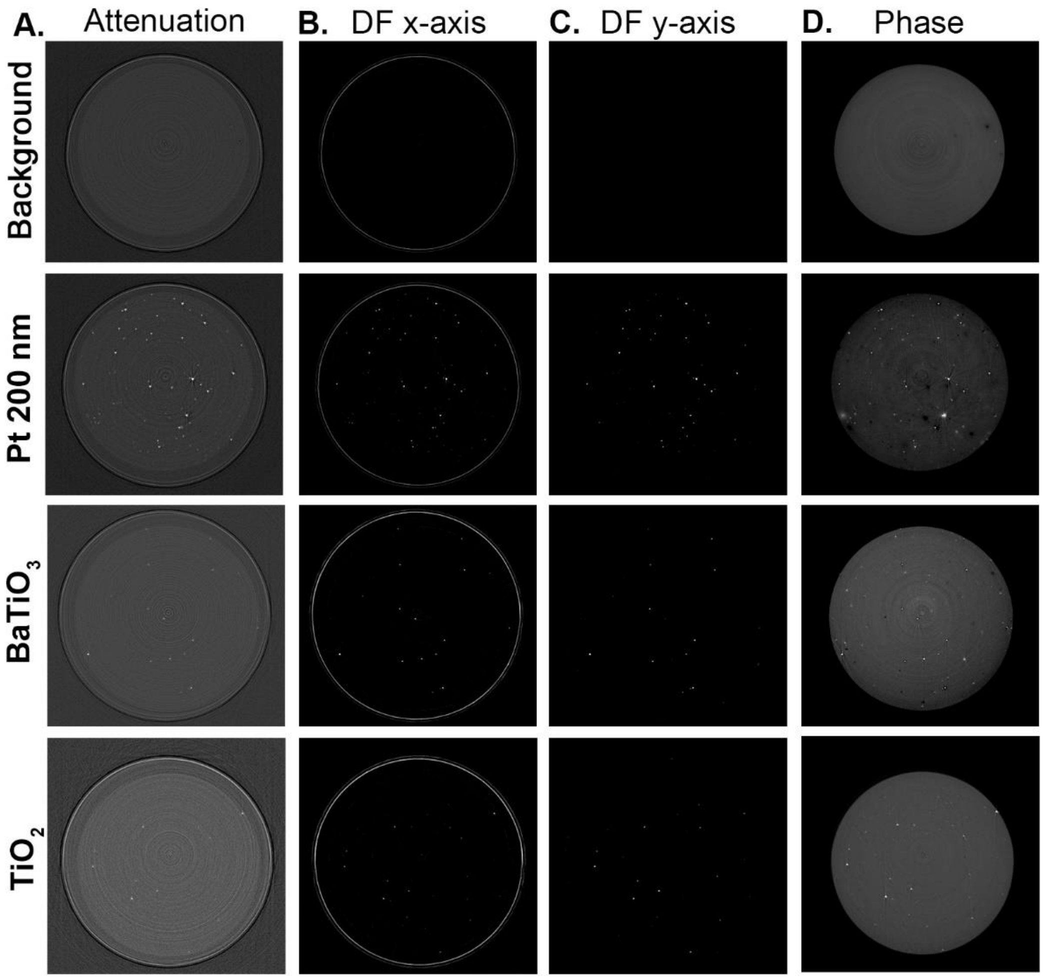

Figure S17. Transverse single slice computed tomographs showing signal from background (1% agar), and 0.5mg/mL nanoparticle concentrations (Platinum nanospheres of 50nm and 200nm diameter, Barium titanate (IV; <100nm), and Titanium dioxide (IV; <100nm) in **A.** Attenuation, **B.** Dark field (x direction), **C.** Dark field (y direction), and **D.** Phase contrast channels.

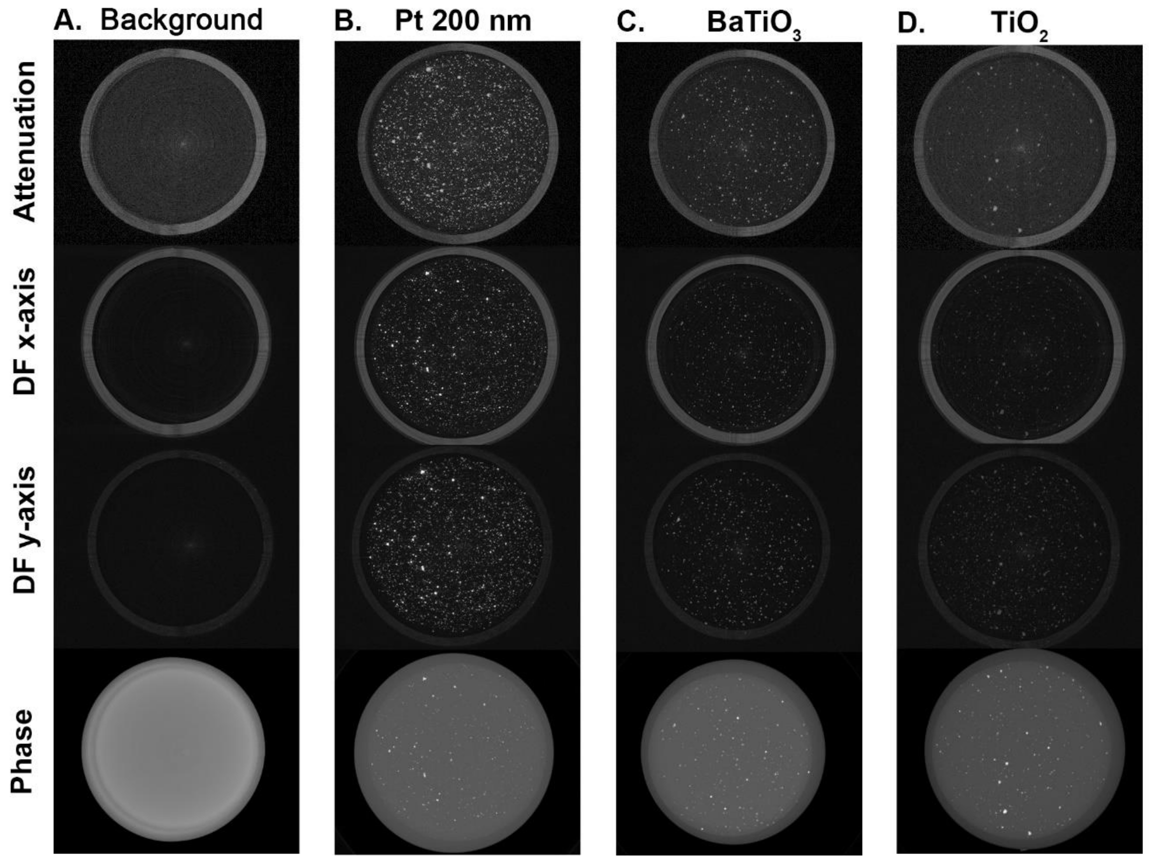

Figure S18. Transverse maximum intensity projection (100 slices) of CT reconstructions in attenuation, dark field (x-axis), dark field (y axis), and phase for **A.** a representative background region of 1% agar only, and 0.5mg/mL concentrations of **B.** Platinum nanopowder (200nm), **C.** $BaTiO_3$, and **D.** TiO_2.

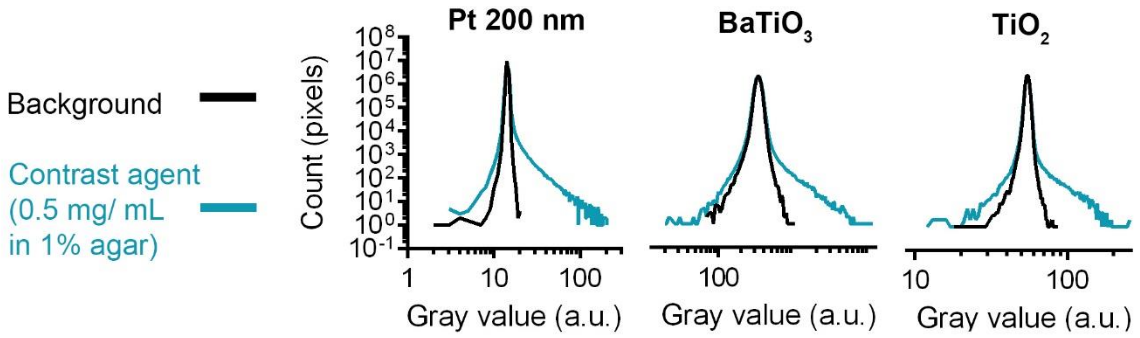

Figure S19. Dark field (y-axis) histogram plots of raw pixelwise grey values for the above nanoparticle types respectively for 100 slices, alongside an equivalent adjacent control (1% agar) region in the same sample.

Particle name	CNR Dark Field	CNR Attenuation
Diatrozoate	0.0886	0.0099
Pt 50 nm	1.94	1.63
Pt 200nm	2.50	2.08
$BaTiO_3$	2.12	1.53

Table S1. Contrast to noise ratios calculated for dark field and attenuation maximum intensity projections, with data acquired using the 1D beam tracking method.

Particle name	CNR Dark Field	CNR Attenuation
Pt 200nm	7.94	5.65
$BaTiO_3$	2.83	1.66
TiO_2	2.87	1.14

Table S2. Contrast to noise ratios calculated for dark field and attenuation maximum intensity projections (100 slices per sample), for data acquired using the 2D beam tracking method.

References

1 Vittoria, F. A. *et al.* Beam tracking approach for single–shot retrieval of absorption, refraction, and dark–field signals with laboratory x–ray sources. *Appl. Phys. Lett.* **106** (2015).

2 Maughan Jones, C. J., Vittoria, F. A., Olivo, A., Endrizzi, M. & Munro, P. R. T. Retrieval of weak x-ray scattering using edge illumination. *Optics letters* **43**, 3874-3877, doi:10.1364/OL.43.003874 (2018).

3 Guizar-Sicairos, M., Thurman, S. T. & Fienup, J. R. Efficient subpixel image registration algorithms. *Optics letters* **33**, 156-158, doi:10.1364/ol.33.000156 (2008).

4 Dreier, E. S. *et al.* Single-shot, omni-directional x-ray scattering imaging with a laboratory source and single-photon localization. *Optics letters* **45**, 1021-1024, doi:10.1364/OL.381420 (2020).

5 Kottler, C., David, C., Pfeiffer, F. & Bunk, O. A two-directional approach for grating based differential phase contrast imaging using hard x-rays. *Optics express* **15**, 1175-1181, doi:10.1364/oe.15.001175 (2007).

6 van Aarle, W. *et al.* The ASTRA Toolbox: A platform for advanced algorithm development in electron tomography. *Ultramicroscopy* **157**, 35-47, doi:10.1016/j.ultramic.2015.05.002 (2015).
